# Variations in Facial Nerve Branches and Anatomical Landmarks for Its Trunk Identification: A Pilot Cadaveric Study in the Lithuanian Population

**DOI:** 10.7759/cureus.6100

**Published:** 2019-11-08

**Authors:** Dominykas Stankevicius, Andrej Suchomlinov

**Affiliations:** 1 Department of Anatomy, Histology, and Anthropology, Vilnius University Faculty of Medicine, Vilnius, LTU

**Keywords:** double trunk, facial nerve, facial nerve palsy, parotidectomy, tragal pointer

## Abstract

Objective

The purpose of this study was to evaluate facial nerve (FN) branching variations based on Davis and Kopuz classifications in the Lithuanian population and measure the shortest distance from the facial nerve trunk (FNT) to its anatomical landmarks.

Methods

Twenty-two hemifaces of 11 cadavers were dissected. The preauricular skin cut was made and extended behind the ear lobe and along the inferior border of the mandible. The skin with subcutaneous tissue and superficial fascia were separated and medially retracted, and the parotid gland was dissected anterogradely. The FNT and its furcation type and branching pattern were disclosed and noted based on Davis and Kopuz classifications. Further, the shortest distance from the FNT to the anatomical landmarks of the tragal pointer (TP), the angle of mandible (AM), and the tip of mastoid process (TMP) was measured.

Results

The prevalence of branching patterns did not differ significantly compared to Davis classification. Based on Kopuz, type IVA pattern was the most common in six cases (27%). Eighteen (82%) trunks split as bifurcations and two (9%) trifurcations, while two (9%) had separate double trunks. The shortest distance (mm) from the FNT to the TP is 9.30 ± 0.93, AM 36.45 ± 4.14, and TMP 12.52 ± 2.30.

Conclusion

The prevalence of FN variations in the Lithuanian population is similar to Davis classification. The AM and TMP are consistent superficial bony landmarks for trunk identification, while the distance from the TP highly varies among studies. Surgeons should be aware of double FNT during parotidectomy, which is described in Kopuz classification.

## Introduction

The facial nerve (FN) is the VII^th^ cranial nerve that controls mimic muscles and is responsible for facial expression. The trunk of FN emerges from stylomastoid foramen and passes through the parotid gland, and it usually bifurcates into upper (temporofacial) and lower (cervicofacial) divisions, both of which further give rise to five terminal branches: temporal, zygomatic, buccal, marginal mandibular, and cervical.

The branching of FN was first described by Davis and colleagues in 1956 [1]. They outlined six FN types (I, II, III, IV, V, and VI) with their recurrence, based on the presence or absence of the anastomoses between terminal branches. This classification is still widely used as a classic pattern in the scientific literature. However, a newer classification was released in 1987 by Katz and Catalano, which did not fit Davis classification [2]. It had nine types of branching patterns (IA, IB, II, IIIA, IIIB, IIIC, IVA, IVB, and V) and was based on anastomoses between terminal branches, the origin of the buccal branch, and the number of FNTs. The improved version with three additional “double-trunk” types (VA, VB, VC) was released in 1994 by Kopuz et al. [3].

The parotidectomy is a well-recognized and effective surgical procedure to treat benign and malignant tumors of the parotid gland. Neoplasms located in the superficial lobe can be treated by partial parotidectomy, whereas lesions extending or arising from the deep lobe require total parotidectomy. However, one of the most common postoperative complications is FN weakness [4-6]. In most benign tumor treatment cases, the FN weakness is temporary, and full recovery is usually achieved within six months after the surgery [7-8]. According to P. Wolber and colleagues, for 40.2% of patients who had undergone superficial parotidectomy, the FN dysfunction developed on the first day and for 14%, it persisted after six months post-surgery [9]. Fortunately, the permanent FN paralysis after parotidectomy is far less common. Recently published papers revealed that FN paralysis persists for 0% to 9.0% of patients 12 months post-surgery [5,7-9]. The identification of FNT during parotidectomy is essential to avoid this complication. Some authors have reported soft-tissue and bony landmarks to assist the surgeon for the early identification of this nerve [10-12]. However, there has been much debate in literature defining the safest and most reliable landmark. Since the FN paralysis remains an issue in maxillofacial surgery, it is necessary to understand the anatomy and topography of the FN for performing any surgical intervention in the parotid area of the face.

The present study aimed to evaluate the recurrence of FN branching types based on Davis and Kopuz classifications and count the FNT furcation cases. We also intended to measure the shortest distance from FNT to superficially located and often used anatomical landmarks: tragal pointer (TP), the tip of mastoid process (TMP), and the angle of mandible (AM).

## Materials and methods

This study was performed at the Vilnius University Faculty of Medicine, Lithuania. Eleven adult Lithuanian cadavers (seven female and four male) donated to the Department of Anatomy, Histology, and Anthropology were used in this study. All bodies were embalmed with formaldehyde 10% solution. Both sides (22 hemifaces) for all corpses were dissected in a semilateral position. A preauricular skin cut was made and then extended behind the ear lobe, over the mastoid process, and along the inferior border of the mandible (Modified Blairs incision) [13]. Skin with subcutaneous tissue and superficial fascia were separated and medially retracted to expose the parotid gland. Further, the parotid gland was carefully dissected from the posterior portion until the FNT was identified and all terminal branches of FN were disclosed. The entire trunk was exposed from the emergent in stylomastoid foramen until its furcation point. The upper part of the sternocleidomastoid muscle was cut and retracted to reveal the bony TMP. The incision in the masseter muscle was made until the AM was fully exposed. Further, the branching pattern of FN was photographed, and schematic illustrations were drawn.

**Figure 1:**
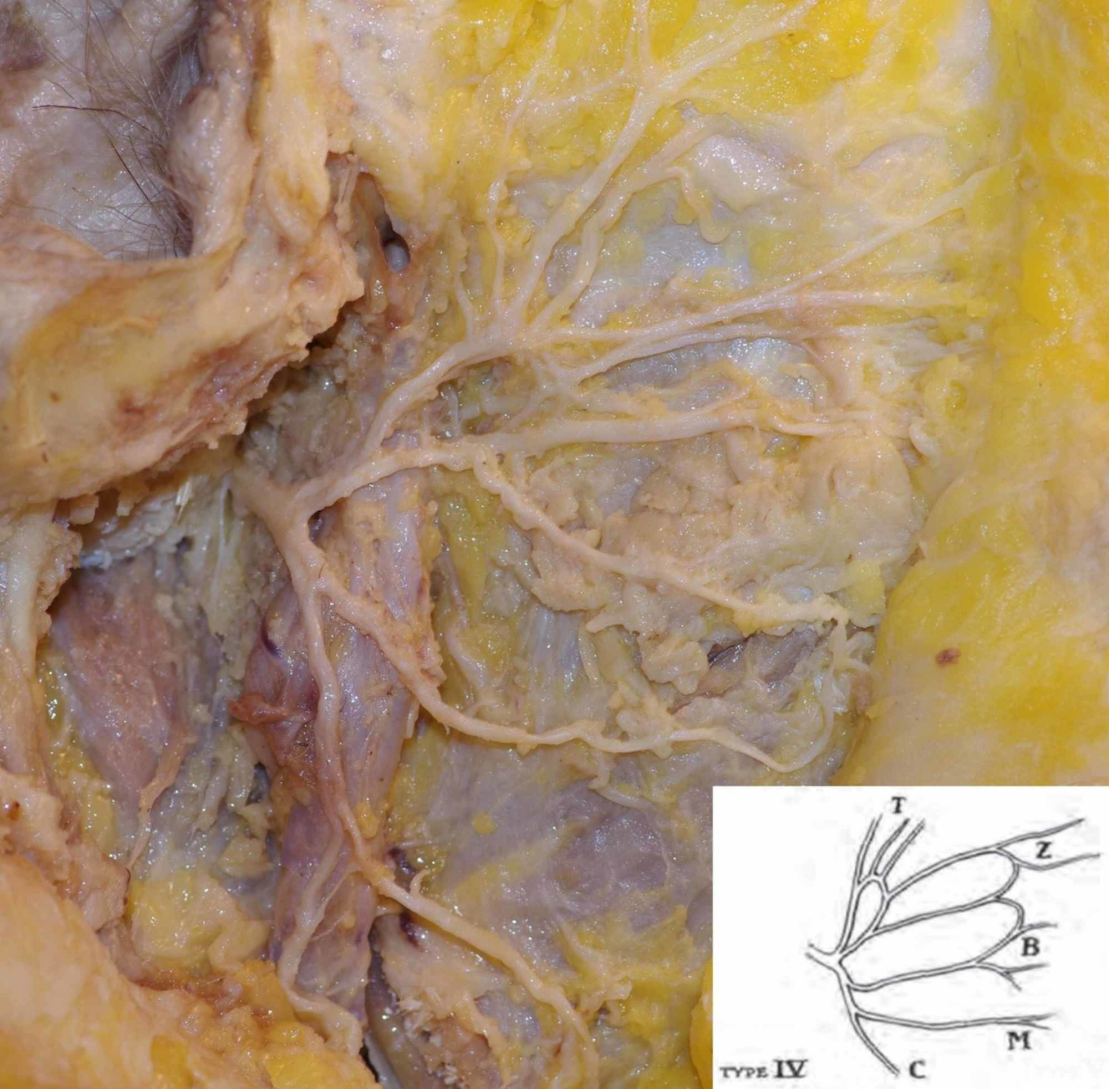
Bifurcation of a single facial nerve trunk; type IV branching pattern based on Davis classification

This study consisted of two investigations. Firstly, we investigated the anatomy of the facial nerve: the branching patterns based on Davis and Kopuz classifications and the furcation types of the trunk (bifurcation, trifurcation or double trunks) were noted (Figure 1).

**Figure 2:**
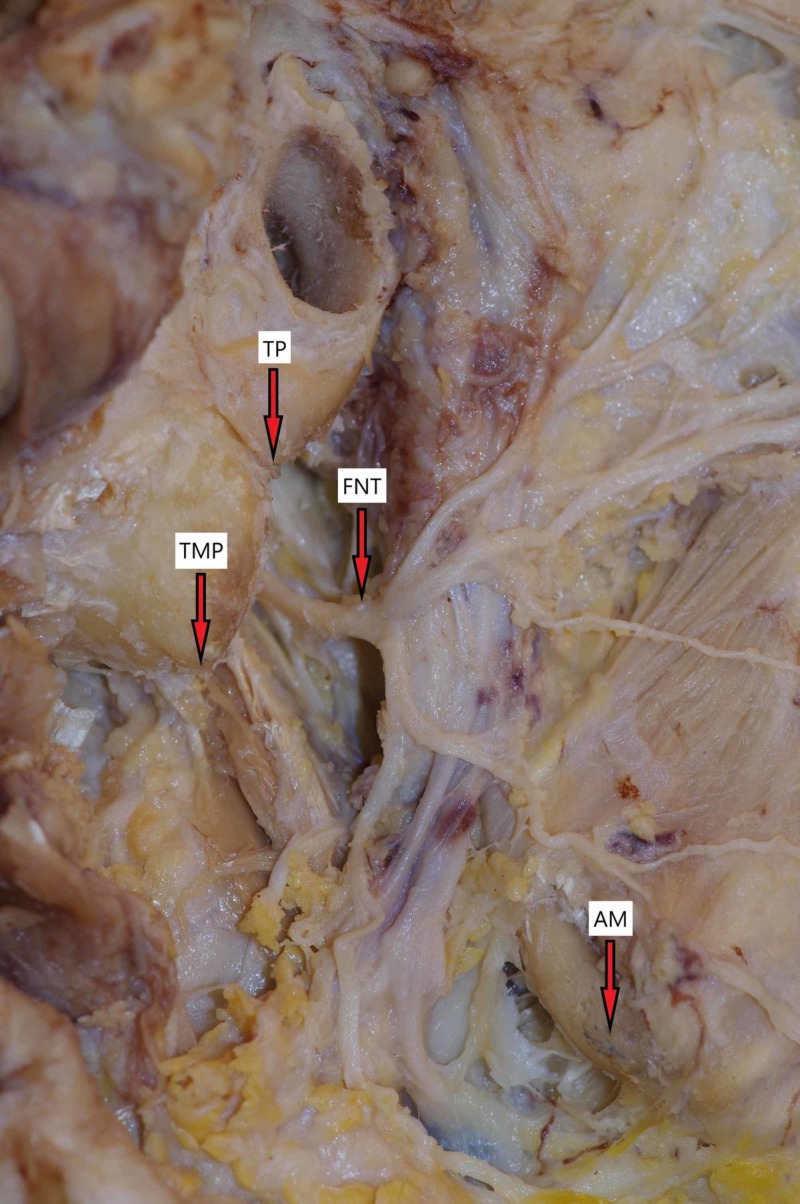
Facial nerve and anatomical landmarks TMP, tip of mastoid process; TP, tragal pointer; AM, angle of mandible; FNT, facial nerve trunk

Secondly, the morphometric measurements were made. We calculated the shortest distance from the FNT to the anatomical landmarks: TP, TPM, and AM (Figure 2).

All the anatomical points were marked with a marker, and measurements were made five times by a single researcher using a digital caliper capable of measuring the lowest value of 0.01 mm. FNs with separate double trunks (two out of 22 cases) were not morphometrically measured. The mean values were calculated using MS Excel 365, and statistical analysis was performed with IBM SPSS Statistics 23 software. The normality of all values was tested using Kolmogorov-Smirnov and Shapiro-Wilk tests. Non-parametrical Mann-Whitney U test was used to compute the statistically significant difference between gender/ furcation type/ side of FN and the mean values of all morphometric measurements. Non-parametrical Kruskal-Wallis test was used to count the statistically significant difference among FN types (Davis and Kopuz classifications) and mean values of morphometric measurements. Fisher’s exact test was performed to count the statistical difference between branching patterns recurrence of this study compared to Davis and Kopuz classification. The level of significance (*p*) was chosen to be less than 0.05.

## Results

After assigning each facial nerve to Davis's branching patterns, the results were as follows: Types I, V, and VI were the least common with two cases in each (9%), type II had three cases (14%), and type III and IV were the most common with seven and six cases, respectively (32% and 27%). We have noticed the familiar percentage results empirically comparing Davis classification to this study (Table 1). 

**Table 1:** Facial nerve branching pattern types recurrence based on Davis classification [1]

Study	N	I	II	III	IV	V	VI
Davis et al. (1956)	350	44 (13%)	71 (20%)	99 (28%)	82 (24%)	32 (9%)	22 (6%)
Present study	22	2 (9%)	3 (14%)	7 (32%)	6 (27%)	2 (9%)	2 (9%)

The branching patterns in this study, based on Kopuz classification, revealed that types IVA and IVB were the most common with six and four cases (27% and 18%), while types IB, II, and VC had no cases to be assigned to (Table 2).

**Table 2:** Facial nerve branching pattern types recurrence based on Kopuz classification [3]

Pattern type	N	IA	IB	II	IIIA	IIIB	IIIC	IVA	IVB	VA	VB	VC
Kopuz et al. (1994)	50	6 (12%)	6 (12%)	6 (12%)	3 (6%)	1 (2%)	3 (6%)	1 (2%)	18 (36%)	3 (6%)	1 (2%)	2 (4%)
Present study	22	3 (14%)	0 (0%)	0 (0%)	2 (9%)	1 (5%)	3 (14%)	6 (27%)	4 (18%)	1 (5%)	2 (9%)	0 (0%)

The difference between the recurrence of Kopuz’s IVA branching pattern type (one case) compared to this study (six cases) appeared to be statistically significant (*p *= 0.003). No statistically significant difference was found among Davis and Kopuz branching patterns compared to morphometric measurements of FNT - TP; FNT - AM; FNT - TMP (Davis: *p *= 0.149; 0.640; 0.901, Kopuz: *p *= 0.380; 0.349; 0.530).

The mean values of the morphometric measurements were as follows: · FNT - TP: 9.30 ± 0.93mm (min-max: 7.67-10.78 mm); · FNT - AM: 36.45 ± 4.14mm (min-max: 25.84-44.39 mm); · FNT - TMP: 12.53 ± 2.30mm (min-max: 8.99-17.26 mm; Table 3).

**Table 3:** Morphometric measurements on male, female, and both gender cadavers The mean values are shown with standard deviation (SD). FNT, facial nerve trunk; TP, tragal pointer; AM, angle of mandible; TMP, tip of mastoid process

Morphometric measurement (distance)	Gender	N	Min-max values (mm)	Mean value ± SD (mm)	P-value
FNT – TP	Male	7	9.41-10.78	10.06 ± 0.59	0.005
	Female	13	7.67-10.10	8.89 ± 0.83	
	Both	20	7.67-10.78	9.30 ± 0.93	
FNT – AM	Male	7	34.11-44.39	39.18 ± 3.43	0.030
	Female	13	25.84-40.50	34.97 ± 3.81	
	Both	20	25.84-44.39	36.45 ± 4.14	
FNT – TMP	Male	7	8.99-16.08	11.77 ± 2.39	0.275
	Female	13	10.58-17.26	12.93 ± 2.25	
	Both	20	8.99-17.26	12.53 ± 2.30	

Out of the studied 22 FNTs, 18 (82%) split as bifurcation and two as trifurcation (9%), while two cases had separate double trunks (9%). No statistically significant difference was found between the FN furcation type and the morphometric measurements (*p* = 0.316; 0.853; 0.263). The differences between the side of FN and morphometric measurements were not statistically significant as well (*p *= 1.000; 0.824; 0.941). However, we have found a statistically significant association between gender and morphometric measurements. The mean value of FNT - TP in male cadavers was significantly higher compared to female cadavers, 10.06 ± 0.59 and 8.89 ± 0.83, respectively (*p *= 0.005). The mean value of FNT - AM distance was 39.18 ± 3.43 mm in male cadavers and 34.97 ± 3.81 mm in female cadavers, and the difference was statistically significant as well (*p *= 0.030; Table 3).

## Discussion

Even though many studies describe the anatomy of the FN and its trunk, FN palsy remains a common complication post-surgery of parotidectomy. Early identification of FNT is essential in preserving the mimic function and facial expression. Various anatomical landmarks (the posterior belly of the digastric muscle, external auditory canal, styloid process, tympanomastoid suture, etc.) were studied and described in the anatomic and surgical literature to assist the surgeon in recognizing this nerve. However, there is still much debate about the safest and most reliable landmark. According to Saha S et al., the posterior belly of the digastric muscle is the best landmark for the identification of the FNT [10]. Shawn T. Joseph and colleagues concluded that the styloid base and the origin of the posterior belly of digastric are safe bony landmarks in FNT identification [14]. We believe that the best landmarks for early identification of FNT are those that are superficially located, can be palpated easily, and do not require deep and complex tissue dissection. This idea is also supported by the article of N. Pather and M. Osman [12]. The TP, AM, and TMP fit all these criteria and were chosen in this study. In the research of N. Pather and M. Osman where 40 cadaveric specimens were dissected, the mean distance of FNT - AM was 38.10 ± 3.10 mm [12]. Our results were not much different: 36.45 ± 4.14 mm. The distance of FNT - TMP was measured by Farahvash et al. They described the mean values on both head sides: 11.81 ± 2.01 on the right and 11.62 ± 1.93 on the left [15]. We got similar numbers: 12.52 ± 2.30 mm. In another study conducted by Borle M et al., an anatomical Borle’s triangle was outlined [11]. Two straight lines were drawn alongside the posterior ramus of the mandible and posterior belly of the digastric muscle, forming the apex of the triangle. The third line starting from TMP which connects previous lines forms the base of the triangle. According to the authors, the FNT is found inside the triangle with a mean distance of 12.18 ± 2.00 mm from TMP [11]. This value appears to be very similar to this study as well (12.52 ± 2.30 mm). The comparison of these results leads to the fact that AM and TMP are anatomically consistent and can be reliably used for maxillofacial surgeons in FNT identification.

The TP is an anatomical landmark, which is used for FNT identification as it usually “points” straight to FNT and is in the distance of around one centimeter from it. However, the axiom that FNT is located 1-1.5 cm to TP was denied by C. Ron Cannon and coauthors [16]. They measured significantly lower distance than the previously accepted standards. For comparison, in this study, the mean FNT - TP distance was found to be 9.30 ± 0.93mm. During literature analysis, we have noticed that the distance of FNT - TP highly varies among studies (Table 4).

**Table 4:** Distance from FNT to tragal pointer measured in other studies Values are shown as means with standard deviation or with min-max values. FNT, facial nerve trunk; TP, tragal pointer [10], [12], [16-20]

Study	FNT – TP (mm)
Saha et al.	16.61 (14-21)
Pather and Osman	34 (24.3-49.2)
Cannon et al.	6.37 (5.84-6.89)
Wong	18.6 ± 6.0
De Ru et al.	8.4 ± 3.6 (observer 1), 7.3 ± 2.4 (observer 2)
Rea et al.	6.9 ± 1.8
Ullah et al.	19.12 (16.5-21.5)
Present study	9.30 ± 0.93

Very different and questionable results lead to hesitations if the “pointer” of tragal cartilage is interpreted equally by anatomists and surgeons, since the cartilage is anatomically “big”, and the measurement point can be marked anywhere. For this reason, we believe that the TP cannot be considered as a reliable and anatomically consistent landmark in the identification of FNT.

The statistical calculations have shown that the mean distances of FNT - TP and FNT - AM are significantly greater in male cadavers compared to female ones (Table 3). A statistically significant difference (*p *= 0.00) of the distance from FNT to AM between male and female cadavers was also noticed by Pather and Osman [12]. We presume that these differences may be related to anatomical variability between males and females since males have more prominent mandibles. We have not found any articles concluding statistically significant FNT - TP distance between males and females.

There are some studies analyzing branching patterns based on popular Davis’ classification in different populations. The data comparison of branching recurrence showed that type III remains the most common pattern in North American, Malaysian, and South Korean populations [1,21-24]. In the present study, regardless of the lower number of studied subjects, type III was the most common as well (32%). However, the study conducted by Weerapanta et al. showed that type V is the most frequent in the Thailand population [25]. Thuku et al. concluded type I as the most common in the African population [26]. Rana et al., Quadros et al., and Bendella & colleagues found type II as the most common in Pakistan, Indian, and German subjects, respectively [27-29] (Table 5).

**Table 5:** Facial nerve branching pattern types recurrence based on Davis classification in different studies [1], [21-29]

Study	N	Population	I	II	III	IV	V	VI
Davis et al.	350	North American	44 (13%)	71 (20%)	99 (28%)	82 (24%)	32 (9%)	22 (6%)
Bernstein and Nelson	35	North American	(9%)	(9%)	(25%)	(19%)	(25%)	(16%)
Myint et al.	79	Malaysian	9 (11.4%)	12 (15.2%)	27 (34.2%)	15 (19%)	6 (7.6%)	10 (12.7%)
Park and Lee	111	South Korean	7 (6.3%)	15 (13.5%)	37 (33.45%)	26 (23.4%)	7 (6.3%)	19 (17.1%)
Lee at al.	41	South Korean	2 (5%)	10 (24%)	14 (34%)	8 (20%)	5 (12%)	2 (5%)
Weerapanta et al.	100	Thailand	1 (1%)	10 (10%)	20 (20%)	18 (18%)	29 (29%)	21 (21%)
Thuku et al.	40	African	10 (25%)	9 (22.5%)	7 (17.5%)	6 (15%)	2 (5%)	6 (15%)
Rana et al.	100	Pakistan	9 (9%)	39 (39%)	20 (20%)	25 (25%)	6 (6%)	1 (1%)
Quadros et al.	20	Indian	2 (10%)	15 (75%)	1 (5%)	1 (5%)	1 (5%)	1 (5%)
Bendella et al.	95	German	39 (24.7%)	40 (25.3%)	31 (19.6%)	19 (12%)	18 (11.4%)	11 (7%)
Present study	22	Lithuanian	2 (9%)	3 (14%)	7 (32%)	6 (27%)	2 (9%)	2 (9%)

**Figure 3:**
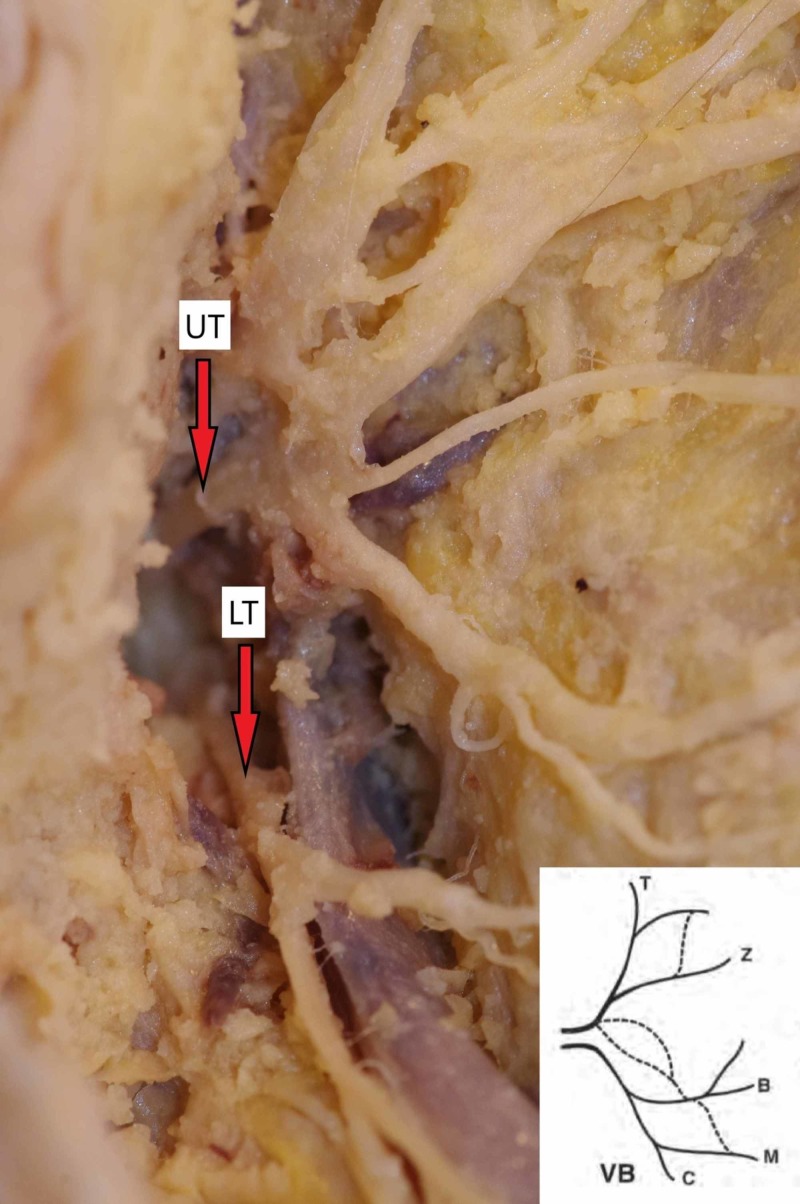
Two separate trunks of the facial nerve emerging from the skull base Type VB pattern based on Kopuz classification UT, upper trunk; LT, lower trunk

It is clearly seen that percentage varies among studies and we believe the recurrence of branching pattern types may be determined by population features. Nonetheless, we have not found any articles describing FN branching dependency on specific genes or environmental factors. It is important to emphasize that a relevant feature of “double-trunk” FN was not described by Davis in his classification. Based on the research, the phenomenon of separate FNT is rare: according to Katz and Catalano, three cases (3%) had separate two main trunks while Kopuz reported only one case (2%) [2-3]. We have not found any other articles describing the recurrence of the FN with separate main trunks. However, in this paper, even two (9%) out of 22 studied facial nerves had separate two main trunks emerging from the skull base (Figure 3).

The anatomy of this variation was described by Botman and Jongkees [30]. They reported that FN can split into two or three trunks within the mastoid segment that further exits through a separate osseous foramen. We suppose this FNT variation is clinically important; therefore, surgeons should always keep in mind the possibility of a “double trunk” FN to avoid injuring it.

## Conclusions

The knowledge of FN and its trunk anatomy and topography is essential in performing successful parotid gland surgery. The branching patterns recurrence in the Lithuanian population does not significantly differ from the popular Davis classification. Two separate main trunks emerging from the skull base can be a clinically significant pattern, which is described by Kopuz’s classification. Therefore, surgeons should keep this possibility in mind and take precautionary measures to avoid the injury. Compared to the other studies, superficially located bony landmarks such as the AM and TMP are anatomically consistent and can be reliably used by surgeons for FNT identification. The cartilaginous TP, on the other hand, is a debatable landmark since the distance greatly varies among studies.
